# Lack of Helios During Neural Development Induces Adult Schizophrenia-Like Behaviors Associated With Aberrant Levels of the TRIF-Recruiter Protein WDFY1

**DOI:** 10.3389/fncel.2020.00093

**Published:** 2020-05-14

**Authors:** Anna Sancho-Balsells, Veronica Brito, Belissa Fernández, Mónica Pardo, Marco Straccia, Silvia Ginés, Jordi Alberch, Isabel Hernández, Belén Arranz, Josep M. Canals, Albert Giralt

**Affiliations:** ^1^Departament de Biomedicina, Facultat de Medicina i Ciències de la Salut, Universitat de Barcelona, Barcelona, Spain; ^2^Institut de Neurociències, Universitat de Barcelona, Barcelona, Spain; ^3^Institut d’Investigacions Biomèdiques August Pi i Sunyer (IDIBAPS), Barcelona, Spain; ^4^Centro de Investigación Biomédica en Red sobre Enfermedades Neurodegenerativas (CIBERNED), Madrid, Spain; ^5^Laboratory of Stem Cells and Regenerative Medicine, Department of Biomedical Sciences, Faculty of Medicine and Health Science, University of Barcelona, Barcelona, Spain; ^6^Faculty of Medicine and Health Science, Production and Validation Center of Advanced Therapies (Creatio), University of Barcelona, Barcelona, Spain; ^7^Alzheimer Research Center and Memory Clinic, Fundació ACE, Institut Català de Neurociències Aplicades. Barcelona, Spain; ^8^Parc Sanitari Sant Joan de Déu, CIBERSAM, Barcelona, Spain

**Keywords:** hippocampus, putamen, cortex, FENS1, psychosis, negative symptoms, DISC1

## Abstract

The role of the WDFY1 protein has been studied as a TLR3/4 scaffold/recruiting protein in the immune system and in different oncogenic conditions. However, its function in brain remains poorly understood. We have found that in mice devoid of *Helios* (He^–/–^ mice), a transcription factor specifically expressed during the development of the immune cells and the central nervous system, there is a permanent and sustained increase of *Wdfy1* gene expression in the striatum and hippocampus. Interestingly, we observed that WDFY1 protein levels were also increased in the hippocampus and dorsolateral prefrontal cortex of schizophrenic patients, but not in the hippocampus of Alzheimer’s disease patients with an associated psychotic disorder. Accordingly, young He^–/–^ mice displayed several schizophrenic-like behaviors related to dysfunctions in the striatum and hippocampus. These changes were associated with an increase in spine density in medium spiny neurons (MSNs) and with a decrease in the number and size of PSD-95-positive clusters in the *stratum radiatum* of the CA1. Moreover, these alterations in structural synaptic plasticity were associated with a strong reduction of neuronal NF-κB in the pyramidal layer of the CA1 in He^–/–^ mice. Altogether, our data indicate that alterations involving the molecular axis Helios-WDFY1 in neurons during the development of core brain regions could be relevant for the pathophysiology of neuropsychiatric disorders such as schizophrenia.

## Introduction

Schizophrenia is a chronic debilitating neuropsychiatric disorder affecting approximately 1% of the population worldwide (Saha et al., 2005). Symptoms cluster into three categories: positive (hallucinations, delusions, and agitation), negative (social withdrawal, anhedonia, and poverty of thought), and cognitive (working memory and social cognition deficits) (Kahn et al., 2015). The onset of the disease occurs typically around post-adolescence (Kahn et al., 2015), and it is considered to be a multifactorial neurodevelopmental disorder (van Os et al., 2010). Immunopathogenesis has been proposed as a possible cause of schizophrenia. According to this hypothesis, the aberrant interaction of the immune system with the central nervous system may provoke alterations such as brain inflammation contributing to schizophrenia (Corsi-Zuelli et al., 2017). In support of the immunopathogenesis theory, early infections and other immune alterations during pregnancy and early post-natal life have been shown to contribute to schizophrenia (Cordeiro et al., 2015). Therefore, genetic predisposition together with both immunological and neural alterations during development could trigger schizophrenic symptoms.

The Ikaros family of transcription factors is required for the normal development of lymphocytes and other blood cell lineages and to produce cytokines (Merkenschlager, 2010). This family is comprised of five related factors (Ikaros, Helios, Aiolos, Eos, and Pegasus, or *IKZF1-5*, respectively), which are expressed at different times during B and T cell development (Thornton et al., 2010; Mitchell et al., 2017). Interestingly, we and others have shown that, only during development, Helios is also present in neuronal cell subtypes in the striatum and hippocampus (Martín-Ibáñez et al., 2012, 2017), which are key affected brain regions in schizophrenia patients (Silbersweig et al., 1995; Yoon et al., 2013). Using a high throughput RNAseq approach, we have identified a core Helios target, the *Wdfy1* (WD Repeat and FYVE Domain Containing 1) gene, which is aberrantly upregulated in the hippocampus and striatum of mice devoid of *Helios* (Giralt et al., 2019). The WDFY1 protein is an adaptor protein for the Toll-like receptor 3 and 4 (TLR3/4) signaling pathway and it mediates the innate and adaptative immune responses by recruiting the TIR-domain-containing adaptor (TRIF). Therefore, it could play a role by promoting the innate immunity pathway in neurons by activating molecules such as nuclear factor kappa B (NF-κB) (Hu et al., 2015). Interestingly, TLR3 has been shown to regulate the expression of DISC1 (from *Disrupted in schizophrenia 1*) in neurons (Chen et al., 2017), directly linking this signaling pathway to schizophrenia.

In the present work we show that aberrantly and sustained upregulated levels of the *Wdfy1* gene are a very specific and long-lasting molecular hallmark in mice devoid of *Helios* (He^–/–^ mice) in different brain regions. At cellular level, this increase was localized in apical and basal dendrites of the CA1 pyramidal neurons. Interestingly, He^–/–^ mice showed several schizophrenia-like phenotypes. In this line, WDFY1 protein levels were aberrantly upregulated in several brain regions of human post-mortem samples from patients with schizophrenia but not in samples from Alzheimer’s disease patients with psychosis.

## Materials and Methods

### Animals

For *in vivo* experiments in adult mice (all in a C57BL/6 strain background), we used mice devoid of *IKZF2* (He^–/–^, MGI Cat# 4355175, RRID:MGI:4355175), which is the gene that encodes for the Helios protein. Briefly, Helios-deficient mice were generated by using a targeting vector and recombination strategy where the C-terminal part of Helios exon 7 was replaced by a 1.8 kb floxed PGK-neo-poly(A) cassette between the *Sal*I and *Xba*I sites (Cai et al., 2009). The full *knockout* mice (He^–/–^) were previously verified (Giralt et al., 2019) and obtained from crosses between heterozygous mice (He^±^ × He^±^). Mouse genotyping was performed by polymerase chain reaction (PCR) in tail biopsy samples as described elsewhere (Cai et al., 2009). Mice were housed with access to food and water *ad libitum* in a colony room kept at 19–22°C and 40–60% humidity, under a 12:12 h light/dark cycle. For experiments in “adult” mice, we used 8-week-old males and females (the exact number from each gender is specified in each figure legend) to make their age comparable to the age of schizophrenia onset in humans (adolescence/youth) and because younger mice trend toward behaving in an unpredictable ways (from our records). For the developmental experiments the age is specified in each figure legend. Experimental animals were used in accordance with the ethical guidelines (Declaration of Helsinki and NIH, publication no. 85–23, revised 1985, European Community Guidelines, and approved by the local ethical committee (University of Barcelona, C-136/19).

### Homing Test

Animals were separated from the dam and kept for 30 min in the small electric mat at 28 ± 1°C. Individual pups were then transferred to a Plexiglas arena (40 × 30 × 10 cm), with the floor subdivided by 2.5 × 2.5 cm squares. Wood shavings from the home cage were evenly spread under the wire mesh floor on one side of the arena (20 × 15 cm, goal arena) and the pup was placed close to the wall on the opposite side. The time required for each pup to place both forelimbs within the goal area was recorded (cut-off time 3 min). This behavior was monitored at postnatal days 10 and 14 (P10 and P14).

### Three Chamber Sociability Test

The apparatus consisted of three interconnected lined compartments with open doors. Subject mice were habituated to the central compartment with closed doors for 5 min. After the habituation phase, the doors were removed and subjects were tested in the sociability task, and 10 min later the social memory task was performed to evaluate preference for social novelty for 10 additional minutes. Thus, the sociability task consisted of placing the subject mice in the middle chamber and allowing them to explore for 5 min. The doorways into the two side chambers were obstructed by plastic boxes during this habituation phase. After the habituation period, the plastic boxes were removed and an unfamiliar mouse (stranger 1) was placed in one of the side chambers enclosed in a small, round wire cage that allowed nose contact between the bars but prevented fighting. In the social memory task, a second, unfamiliar mouse was placed in the chamber that had been empty during the first 10 min session (or sociability task). This second stranger (stranger 2) was also enclosed in an identical small wire cage. The test mouse had a choice between the first, already-investigated unfamiliar mouse (stranger 1) and the novel unfamiliar mouse (stranger 2). Time sniffing/exploring each small cage were measured using the SMART junior software (Panlab).

### Impulsivity/Jumping Test

The jumping behavior was evaluated with the use of a round platform (an inverted glass container with a diameter of 13 cm and a height of 20 cm); mice were placed on the top of the platform, and their behavior was video recorded for 15 min. Latency to jump out of the platform was measured (Matsuoka et al., 2005).

### Amphetamine and Apomorphine Treatments

Locomotor activity was measured in an open field and recorded with a video caption system. Animals were placed into the center of an open field arena (a white square arena measuring 40 × 40 × 40 cm in length, width, and height respectively), and left to habituate for 25 min. Dim light intensity was 60 lux throughout the arena. After this period, mice received an intraperitoneal injection of vehicle (0.9% NaCl), R-(-)-apomorphine (0.5 mg/kg; SIGMA A4393), or D-amphetamine sulfate (3 mg/kg; TOCRIS 2813) as previously described (Pineda et al., 2005; Sherrill et al., 2013) and their behavior was recorded over the following 45 min. The same mice were evaluated with both dopaminergic stimulants. Data caption were recorded at 1 min intervals using the SMART Junior Software (Panlab) and the distance covered/pathlength (in cm) was analyzed.

### Tissue Fixation and Immunofluorescence

Mice were euthanized by cervical dislocation. Left hemispheres were removed and fixed for 72 h in paraformaldehyde solution (4% in a phosphate buffer 0.1M). 40 μm coronal sections were obtained using a Leica Vibratome (Leica VT1000S). Next, free-floating sections were washed three times in PBS, treated with NHCl for 30 min, and washed again three times with PBS. Floating sections were permeabilized in PBST (0.3%) with 0.02% Azide, 2% BSA, and 3% NGS (Ab buffer) for 1 h at room temperature. After three washes in PBS, brain slices were incubated overnight at 4°C with WDFY1 rabbit 1:100 (Bioss Abs, bs-13169R) or with NF-κB rabbit 1:200 (Santa Cruz Biotechnology Cat# sc-372, RRID:AB_632037). Sections were then washed three times and incubated for 2 h at room temperature with fluorescent secondary antibody AlexaFluor 488 goat anti-rabbit (1:400; from Jackson ImmunoResearch, West Grove, PA, United States). Immunofluorescence specificity was confirmed in slices without primary antibody. Sections were analyzed using a two-photon confocal microscope (Leica SP5).

### DioListic Staining and Spine Counting

Striatal neurons were labeled using the Helios Gene Gun System (Bio-Rad) as previously described (Brito et al., 2014). Briefly, a suspension buffer containing 3 mg of DiI (Molecular Probes, Invitrogen) dissolved in 100 μl of methylene chloride (Sigma-Aldrich) and mixed with 50 mg of tungsten particles (1.7 mm diameter; Bio-Rad) was spread on a glass slide and air-dried. The mixture was resuspended in 3.5 ml distilled water and sonicated. Subsequently, the mixture was drawn into Tefzel tubing (Bio-Rad), and then removed to allow tube drying during 5 min under a nitrogen flow gas. The tube was then cut into 13 mm pieces to be used as gene gun cartridges. Dye-coated particles were delivered in the striatum using the following protocol: shooting was performed over 200 μm coronal sections at 80 psi through a membrane filter of 3 μm pore size and 8 × 10 pores/cm2 (Millipore). Sections were stored at room temperature in PBS for 3 h protected from light and then incubated with DAPI and mounted in Mowiol to be analyzed. DiI-labeled medium-spiny neurons from striatum were imaged using a Leica Confocal SP5 with a × 63 oil-immersion objective (digital zoom 5x). Spine density and morphology analysis was performed using the freeware Neuronstudio (Computational Neurobiology and Imaging Center, Icahn School of Medicine at Mount Sinai) as previously described (de Pins et al., 2019).

### Quantitative (Q)-PCR Assay

Total RNA from hippocampus and striatum in He^+/+^ and He^–/–^ mice at 8 weeks of age was extracted using the Total RNA Isolation Nucleospin^®^ RNA II Kit (Macherey-Nagel, Düren, Germany). Total RNA (500 ng) was used to synthesize cDNA using random primers with the StrataScript^®^ First Strand cDNA Synthesis System (Stratagene, La Jolla, CA, United States). The cDNA synthesis was performed at 42°C for 60 min in a final volume of 20 μl according to manufacturer’s instructions. The cDNA was then analyzed by Q-PCR using the following TaqMan^®^ Gene Expression Assays (Applied Biosystems, Foster City, CA, United States): 18S (Hs99999901_s1), *Ngfr* (Mm00446294_m1), and *Vsnl1* (Hs00374305_m1); or Integrated DNA Technologies (IDT): *Adcy8* (NM_009623), *Aif1* (NM_019467), *Grm2* (NM_001160353), *Kcne4* (NM_021342), *Lancl1* (NM_001190984 and NM_021295), *Pth2r* (NM_139270), and *Wdfy1* (NM_001111279 and NM_001111279). Reverse-transcriptase (RT) polymerase chain reaction was performed in 25 μl volumes on 96-well plates, in a reaction buffer containing 12.5 μl Brilliant^®^ Q-PCR Master Mix (Stratagene), 1.25 μl TaqMan^®^ Gene Expression Assays, and 10–20 ng of cDNA. Reactions were as follows: 40 cycles of a two-step PCR; 95°C for 30 s and 60°C for 1 min, after initial denaturation at 95°C for 10 min. All RT-PCR assays were performed in duplicate and repeated for at least three independent experiments. To provide negative controls and exclude contamination by genomic DNA, the RT was omitted in the cDNA synthesis step, and the samples were subjected to the PCR reaction in the same manner with each TaqMan^®^ Gene Expression Assay. The RT-PCR data were analyzed using the MxProTM Q-PCR analysis software version 3.0 (Stratagene). Quantification was performed with the Comparative Quantitation Analysis program of the mentioned software and using the 18S gene expression as internal loading control.

### Pharmacological Treatments

C57BL/6 male mice (10 weeks old) were injected intraperitoneally with D-amphetamine (3 mg/kg) or with a sub-anesthetic dose of ketamine (30 mg/kg) for 8 days once per day as previously described (Manning et al., 2016; McDougall et al., 2017). On day 8, mice were sacrificed 15 min after injection and the hippocampus, and the striatum were rapidly dissected out and frozen at −80°C for future use in western blot experiments (see *western blot* section). Another group of C57BL/6 male mice at postnatal day 5 were injected intraperitoneally with vehicle or lipopolysaccharides (LPS, 6 mg/kg; Cardoso et al., 2015) or polyinosinic:polycytidylic acid (Poly I:C, 6 mg/kg; Baghel et al., 2018). 24 h later, mice were sacrificed by decapitation and the hippocampus, striatum, and frontal cortex were rapidly dissected out and frozen at −80°C for future use in western blot experiments (see *western blot* section).

### Human Post-mortem Samples

The brain samples from schizophrenia (SZ) patients used in this study were provided by the Sant Joan de Déu Brain Bank (Sant Boi de Llobregat, Barcelona, Spain). The donation and obtaining of samples were regulated by the ethics committee of both institutions. The sample processing followed the rules of the European Consortium of Nervous Tissues: BrainNet Europe II (BNEII). All the samples were protected in terms of individual donor identification following the BNEII laws. Clinical diagnosis of SZ in donor subjects was confirmed premortem with DMS-IV (*Diagnostic and Statistical Manual of Mental Disorders* – 4th edition) and ICD-10 (International Statistical Classification of Diseases and Related Health Problems) criteria by clinical examiners. Most donors were hospitalized for more than 40 years and were re-evaluated every 2 years to monitor and update their clinical progression. Case information can be found in Table 1. Post-mortem samples from humans with Alzheimer’s disease (hippocampus) and controls (hippocampus, putamen, and dorsolateral prefrontal cortex) were obtained from Banc de Teixits Neurològics (Servei Científico-Tècnics, Universitat de Barcelona, Barcelona, Spain) and the sample processing also followed the BNEII rules. Case information can be found in Table 2. To distinguish between Alzheimer’s patients with psychotic symptoms from those without psychotic symptoms, the NIA-AA clinical criteria was used in patients considering the patients score in delusions and/or hallucinations of NPI-Q gravity ≥ 2 (rank from 0 to 3). All the procedures for the obtention of post-mortem samples followed the ethical guidelines of the Declaration of Helsinki and local ethical committees (Universitat de Barcelona: IRB00003099; Fundació CEIC Sant Joan de Déu: BTN-PSSJD).

**TABLE 1:** Demographics, treatments, and illness details for human post-mortem samples from patients with schizophrenia and controls.

Case ID	Diagnostic	Gender	Age (years)	PMI (h)	Medication
1557	Control	M	86	7	NA
1679	Control	F	90	14	NA
1694	Control	M	58	5	NA
1752	Control	F	88	24	NA
1818	Control	M	78	5	NA
1858	Control	F	83	7	NA
1870	Control	F	97	7	NA
1888	Control	F	93	6	NA
1937	Control	F	83	8	NA
1949	Control	M	86	8	NA
0810	Control	F	81	23	NA
1074	Control	M	31	17	NA
0364	Control	F	68	13	NA
0034	Control	M	64	4	NA
0839	Control	M	56	4	NA
0162	Control	F	71	8	NA
0024	Control	F	60	15	NA
0044	Control	M	39	4	NA
1491	Control	M	83	13	NA
1563	Control	M	79	5	NA
1570	Control	F	86	4	NA
1697	Control	M	78	6	NA
1733	Control	M	76	11	NA
1774	Control	F	74	5	NA
P_001	Chronic residual schizophrenia	M	34	2	NA
P_003	Chronic residual schizophrenia	M	80	6	NA
P_008	Chronic residual schizophrenia	M	74	7	Levomepromazine 75 mg/day Haloperidol 15 mg/day
P_011	Chronic residual schizophrenia	M	76	7	Quetiapine 250 mg/day
P_017	Chronic paranoid schizophrenia	M	69	8	Quetiapine 500 mg/day
P_019	Chronic residual schizophrenia	M	79	3	Levomepromazine 13 mg/day, Quetiapine 600 mg/day
P_020	Chronic residual schizophrenia	M	65	4	Quetiapine 1000 mg/day, Amisulpride 800 mg/day
P_021	Schizoaffective disorder	M	82	4	Haloperidol 15 mg/day, Olanzapine 20 mg/day
P_022	Chronic residual schizophrenia	M	79	9	Perphenazine 40 mg/day, Thioridazine 100 mg/day, Sulpiride 200 mg/day, Haloperidol: 10 mg/day
P_025	Chronic residual schizophrenia	M	98	8	NA
P_026	Chronic residual schizophrenia	M	77	6	Olanzapine 20 mg/day
P_027	Chronic residual schizophrenia	M	84	3	Olanzapine 15 mg/day
P_028	Chronic residual schizophrenia	M	79	3	NA
P_029	Chronic residual schizophrenia	M	80	5	Olanzapine 15 mg/day
P_032	Chronic residual schizophrenia	M	92	2	Quetiapine 100 mg/day
P_033	Chronic residual schizophrenia	M	87	4	Olanzapine 5 mg/day
P_034	Chronic residual schizophrenia	M	93	3	NA
P_035	Chronic schizophrenia. Paranoid type	M	86	1	Levomepromazine 5 mg/day
P_037	Chronic residual schizophrenia	M	74	9	Levomepromazine 50 mg/day, Olanzapine 10 mg/day
P_038	Chronic residual schizophrenia	M	75	6	Olanzapine 10 mg/day
P_041	Delusional disorder – Erotomaniac type	F	81	5	NA
P_042	Chronic residual schizophrenia	M	77	2	Risperidone 2 mg/day, Quetiapine 1000 mg/day
P_044	Chronic disorganized schizophrenia	M	78	8	NA
P_045	Chronic residual schizophrenia	F	86	2	Haloperidol 15 mg/day
P_046	Chronic residual schizophrenia	M	82	6	Risperidone 2 mg/day
P_049	Chronic residual schizophrenia	M	79	7	Haloperidol 3 mg/day
P_052	Chronic residual schizophrenia	M	81	6	NA
P_053	Schizophrenia. Paranoid type. In remission	M	80	2	NA
P_055	Chronic residual schizophrenia	M	76	5	Sulpiride 200 mg/day
P_056	Chronic residual schizophrenia	M	83	4	Quetiapine 50 mg/day
P_058	Chronic residual schizophrenia	M	87	9	NA
P_059	Chronic residual schizophrenia	M	83	4	Chlorpromazine 200 mg/day, Haloperidol 10 mg/day, Olanzapine 20 mg/day, Levomepromazine 100 mg/day, Perphenazine 30 mg/day, Tioproperazine 20 mg/day,
P_062	Chronic residual schizophrenia	M	81	8	NA
P_064	Chronic residual schizophrenia	M	89	1	NA
P_065	Chronic residual schizophrenia	M	76	8	Perphenazine 4 mg/day, Olanzapine 5 mg/day
P_068	Chronic residual schizophrenia	M	90	3	NA
P_069	Chronic residual schizophrenia	M	86	6	NA
P_072	Chronic residual schizophrenia	M	83	6	NA
P_074	Chronic residual schizophrenia	M	85	2	Haloperidol 2 mg/day
P_076	Chronic residual schizophrenia	M	75	6	Levomepromazine 25 mg/day, Olanzapine 10 mg/day
P_077	Chronic disorganized schizophrenia	M	84	3	Amisulpride 200 mg/day
P_080	Chronic residual schizophrenia	M	81	2	Zyprexa 10 mg/day, Haloperidol 3 mg/day; Olanzapine 5 mg/day, Tioridazine 150 mg/day,
P_084	Chronic schizophrenia. Catatonic type	M	71	8	Quetiapine 300 mg/day, Olanzapine 40 mg/day
P_086	Chronic schizophrenia. Catatonic type	M	82	7	NA
P_087	Chronic residual schizophrenia	M	74	4	Quetiapine 350 mg/day, Haloperidol 5 mg/day,
P_089	Chronic residual schizophrenia	M	67	7	Olanzapine 10 mg/day, Quetiapine 75 mg/day, Haloperidol 20 mg/day
P_091	Chronic residual schizophrenia a	M	91	3	NA
P_092	Chronic residual schizophrenia	M	78	6	Haloperidol 1 mg/day
P_093	Chronic residual schizophrenia	M	69	7	Haloperidol 15 mg/day, Risperidone 12 mg/day, Olanzapine 30 mg/day
P_096	Schizophrenia. Simple type	M	58	2	Haloperidol 15 mg/day
P_097	Chronic residual schizophrenia	M	50	2	Haloperidol 15 mg/day
P_100	Chronic residual schizophrenia	M	69	3	NA
P_102	Chronic schizophrenia. Paranoid type	M	64	3	Olanzapine 20 mg/day

*PMI, Post-mortem interval; M, Male; F, Female; NA, Not applicable.*

**TABLE 2:** Demographics and illness details for human post-mortem hippocampal samples from patients with Alzheimer’s disease and controls.

Case ID	Diagnostic	Gender	Age (years)	PMI (h)	Medication
1557	Control	M	86	7	NA
1679	Control	F	90	13	NA
1694	Control	M	58	5	NA
1752	Control	F	88	24	NA
1818	Control	M	78	5	NA
1870	Control	F	97	7	NA
1888	Control	F	93	5	NA
1949	Control	M	86	7	NA
1127	Alzheimer’s, no psychosis	M	82	8	NA
1785	Alzheimer’s (early onset), no psychosis	F	74	4	NA
1800	Alzheimer’s, no psychosis	M	84	5	NA
1803	Alzheimer’s (cortico-basal dementia), no psychosis	M	72	18	NA
1822	Alzheimer’s, no psychosis	F	82	6	NA
1824	Alzheimer’s, no psychosis	F	84	6	NA
1829	Alzheimer’s, no psychosis	M	83	5	NA
1847	Alzheimer’s, no psychosis	F	75	16	NA
1879	Alzheimer’s (early onset), no psychosis	F	63	10	NA
1882	Alzheimer’s (early onset), no psychosis	M	72	5	NA
1037	Alzheimer’s (early onset) with psychosis	M	56	16	NA
1273	Alzheimer’s (early onset) with psychosis	M	61	8	NA
1321	Alzheimer’s (early onset) with psychosis	M	63	8	NA
1380	Alzheimer’s with psychosis	F	85	10	NA
1386	Alzheimer’s with psychosis	M	81	3	NA
1443	Alzheimer’s (early onset) with psychosis	F	63	6	NA
1444	Alzheimer’s (early onset) with psychosis	M	62	20	NA
1457	Alzheimer’s with psychosis	F	76	13	NA
1614	Alzheimer’s with psychosis	F	85	12	NA
1736	Alzheimer’s with psychosis	F	86	8	NA

*PMI, Post-mortem interval; M, Male; F, Female; NA, Not applicable.*

### Western Blot

Animals were euthanized by cervical dislocation. The hippocampus was dissected out, frozen using CO_2_ pellets, and stored at −80°C until use. Briefly, the tissue was lysed by sonication in 150 ml of lysis buffer (PBS, 10 ml l1 Non-idet P-40, 1 mM PMSF, 10 mg l1 aprotinin, 1 mg l1 leupeptin and 2 mg l1 sodium orthovanadate). After lysis, samples were centrifuged at 12,000 r.p.m. for 15 min. Supernatant proteins (15 mg) from total brain regions extracts were loaded in polyacrylamide gels (SDS–PAGE) at different polyacrylamide concentrations and transferred to nitrocellulose membranes during 1 h. Membranes were blocked in TBS-T (150 mM NaCl, 20 mM Tris-HCl, pH 7.5, 0.5% Tween 20) with 5% non-fat dry milk and 5% BSA. Immunoblots were probed with the following antibodies: anti-WDFY1 rabbit 1:1000 [SIGMA, # HPA050603; RRID:AB_2681188 (specific for humans)], anti-WDFY1 rabbit 1:1000 [Bioss Abs, bs-13169R (for mice)], anti-VSNL1 rabbit 1:1000 (Proteintech, 13919-1-AP; RRID:AB_2215851), and anti-DISC1 (Proteintech Cat# 15500-1-AP, RRID:AB_2230451). All blots were incubated with the primary antibodies overnight at 4°C by shaking in PBS with 0.2 g l-1 sodium azide. After several washes in TBS-T, blots were incubated with anti-rabbit or anti-mouse horseradish-peroxidase-conjugated secondary antibodies (1:2000; Promega). Secondary antibody binding was detected by the enhanced chemiluminescence substrate kit (Santa Cruz Biotechnology). For loading control, a mouse monoclonal antibody for α-tubulin was used (#083M4847V, 1:30,000; Sigma). ImageJ software was used to quantify the different densitometry immunoreactive bands relative to the intensity of the α-tubulin in the same membranes.

### Statistical Analysis

All data are expressed as mean ± SEM. Statistical analysis were performed using the unpaired two-sided Student’s t-test (95% confidence), one-way ANOVA with the Tukey’s as *post hoc* tests, and two-way ANOVA with the Bonferroni’s *post hoc* test as appropriate and indicated in the figure legends. Values of *p* < 0.05 were considered as statistically significant. All experiments in this study were blinded and randomized. All mice bred for the experiments were used for pre-planned experiments and randomized to experimental groups. Data were collected, processed and analyzed randomly. The experimental design and handling of mice were identical across experiments. Littermates were used as controls with multiple litters (three to five) examined per experiments.

## Results

### *Wdfy1* Is the Only Differentially Expressed Gene That Remains Up-Regulated in the Hippocampus and Striatum of Adult He^–/–^ Mice

**FIGURE 1:**
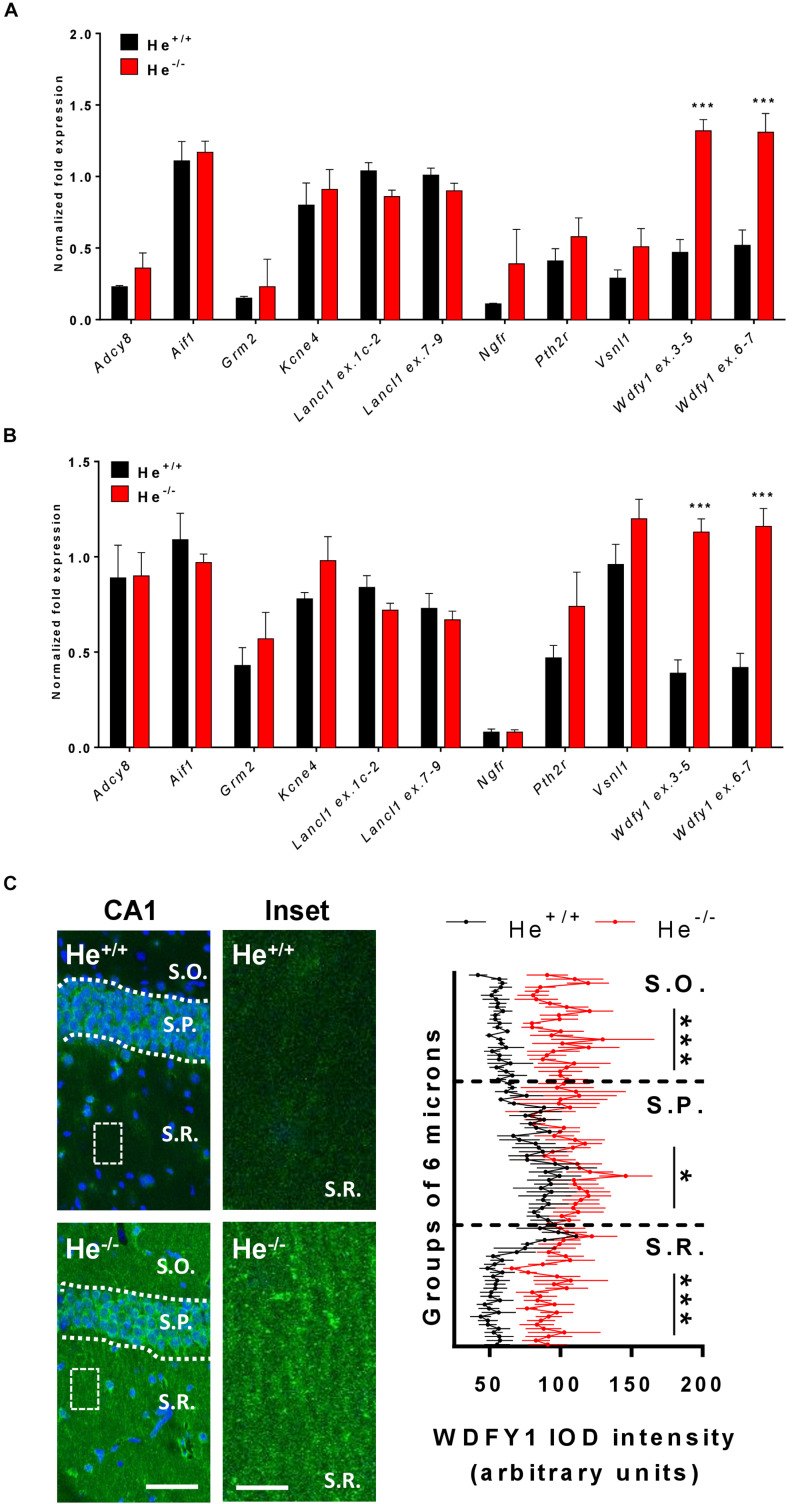
WDFY1 protein and gene expression in He^–/–^ mice. **(A)** mRNA levels of *Adcy8*, *Aif1*, *Grm2*, *Kcne4*, *Lancl1* (two different probes: exons 1c-2 and exons 7–9), *Ngfr*, *Pth2r*, *Vsnl1*, and *Wdfy1* (two different probes: exons 3–5 and exons 6–7) were determined in striatal samples of 8-week-old He^+/+^ and He^–/–^ mice (*n* = 6 He^+/+^ and 6 He^–/–^; 3 males and 3 females per genotype). **(B)** mRNA levels of *Adcy8*, *Aif1*, *Grm2*, *Kcne4*, *Lancl1* (two different probes: exons 1c-2 and exons 7–9), *Ngfr*, *Pth2r*, *Vsnl1*, and *Wdfy1* (two different probes: exons 3–5 and exons 6–7) were determined in hippocampal samples of 8-week-old He^+/+^ and He^–/–^ mice (*n* = 6/genotype). **(C)** Double staining for DAPI (blue) and WDFY1 (green) in the hippocampal CA1 of 8-week-old He^+/+^ and He^–/–^ mice (left). White squares represent inset zones with their magnifications placed at right of each original CA1 image. Quantification (*n* = 2 He^+/+^ males and 2 He^+/+^ females, and 2 He^–/–^ males and 3 He^–/–^ females) of the intensity of the optical density (IOD) in the CA1 is shown separately for *stratum oriens* (S.O.), *stratum pyramidale* (S.P.), and *stratum radiatum* (S.R.). A linear intensity profile analysis as a mean IOD was performed in each image. Points in the X axis indicate mean IOD for each row of pixels. An independent statistical analysis was performed in each CA1 layer. Bars represent mean ± SEM. Data were analyzed by Student’s *t*-test for each gene in **(A,B)** and by two-way analysis of variance (ANOVA) in **(C)**. In **(A),** ***p = 0.000106 and *p* = 0.0008 for *Wdfy1 ex.3–5* and *Wdfy1 ex.6–7* respectively when compared with He^+/+^ mice. In **(B)**, ***p = 1,971446e-005 and *p* = 0.00018 for *Wdfy1 ex.3–5* and *Wdfy1 ex.6–7* respectively when compared with He^+/+^ mice. Scale bar, 70 (left images) and 10 μm (right images).

In a previous study, we carried out a RNAseq experiment at embryonic day 18 (E18) and we identified some genes that were differentially expressed in He^–/–^ mice compared to He^+/+^ mice in both the striatum and the hippocampus (Giralt et al., 2019). Thereby, here we first aimed to verify whether these previously identified genes and others were still dysregulated in the hippocampus and in the striatum of 8-week-old He^–/–^ mice compared to age-matched He^+/+^ mice. All the genes identified to be altered at E18 – *Kcne4*, *Lancl1* (two different probes: exon 1c-2 and exon 7–9), *Pth2r*, and *Vsnl1* – were similarly expressed in 8-week-old He^–/–^ mice compared to He^+/+^ litters in the striatum (Figure 1A) as well as in the hippocampus (Figure 1B). Surprisingly, *Wdfy1* was the only gene that remained up-regulated in 8-week-old He^–/–^ mice compared to controls in both brain regions, striatum (Figure 1A; *t*_10_ = 7.54, *p* < 0.001) and hippocampus (Figure 1B; *t*_10_ = 7.163, *p* < 0.001). Gene expression of *Wdfy1* was further confirmed by using two different probes (exon 3–5 and exon 6–7). We then evaluated the mRNA levels for additional genes. Furthermore, we analyzed the mRNA levels of the *Aif1* gene since it is a very well-known marker of immune activation (macrophage and t-cell activation) (Kelemen and Autieri, 2005). The *Grm2* gene encodes for the mGuR2 receptor, which is a marker of macrophage inflammatory protein 1b and also a crucial regulator of hippocampal synaptic plasticity (Betke et al., 2012), a process that is severely affected in He^–/–^ mice (Giralt et al., 2019). *Adcy8* was used as a marker of the CA1 hippocampal subregion (de Mooij-van Malsen et al., 2009) where the Helios protein was enriched during development (Martín-Ibáñez et al., 2012). In summary, all of these genes were not different between 8-week-old He^–/–^ mice compared to age-matched He^+/+^ mice. Finally, we further validated this genetic *Wdfy1* up-regulation in the CA1 of adult He^–/–^ mice by immunofluorescence (Figure 1C). By using this approach, we observed a significant increase on Wdfy1 protein levels in He^–/–^ mice. This was mostly localized in the *stratum radiatum* [Two-way ANOVA, group effect: *F*_(__1,182__)_ = 106.9, *p* < 0.0001] and in the *stratum oriens* [Two-way ANOVA, group effect: *F*_(__1,252__)_ = 26.11, *p* < 0.0001], but also in the *stratum pyramidale* to a lesser extent [Two-way ANOVA, group effect: *F*_(__1,216__)_ = 5.203, *p* < 0.0105], indicating that, probably, such upregulation was mostly localized in the apical and basal dendrites of the CA1 pyramidal neurons.

### The WDFY1 Protein Is Upregulated in Different Brain Regions of Schizophrenic Patients but Not in Alzheimer’s Disease Patients

**FIGURE 2:**
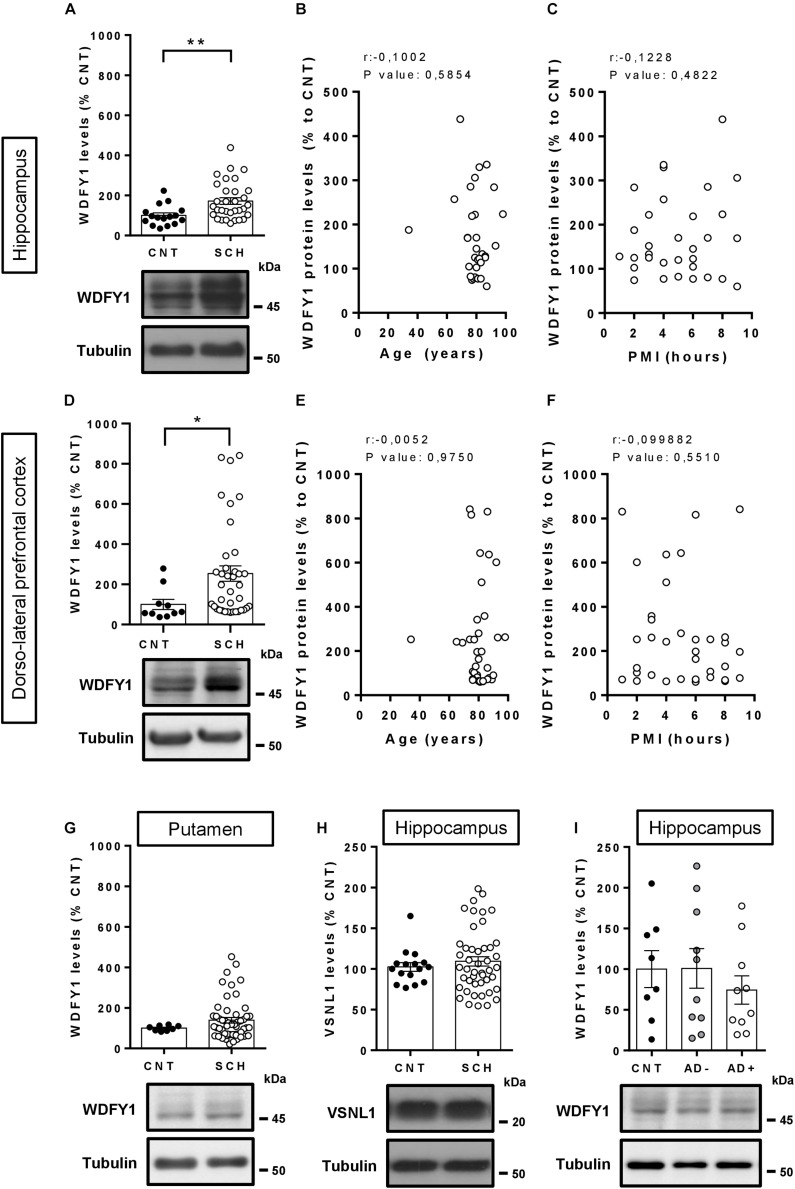
WDFY1 protein levels in patients with schizophrenia and in patients with Alzheimer’s disease with or without psychosis. **(A)** Immunoblotting for WDFY1 and Tubulin as a loading control in the hippocampus of human post-mortem samples from patients with schizophrenia (SCH) and control (CNT) individuals (*n* = 16 CNT and 32 SCH). Correlation analysis comparing WDFY1 protein levels in schizophrenic patients from **(A)** with **(B)** age or **(C)** post-mortem interval (PMI). **(D)** Immunoblotting for WDFY1 and Tubulin as a loading control in the dorsolateral prefrontal cortex of human post-mortem samples from patients with schizophrenia and control individuals (*n* = 10 CNT and 38 SCH). Correlation analysis comparing WDFY1 protein levels in schizophrenic patients from **(D)** with **(E)** age or **(F)** post-mortem interval (PMI). **(G)** Immunoblotting for WDFY1 and Tubulin as a loading control in the putamen of human post-mortem samples from patients with schizophrenia and control individuals (*n* = 8 CNT and 53 SCH). **(H)** Immunoblotting for VSNL1 and Tubulin as a loading control in the hippocampus of human post-mortem samples from patients with schizophrenia and control individuals (*n* = 16 CNT and 46 SCH). **(I)** Immunoblotting for WDFY1 and Tubulin as a loading control in the hippocampus of human post-mortem samples from patients with Alzheimer’s disease with (AD +) or without (AD-) associated/diagnosed psychotic symptoms and control (CNT) individuals (*n* = 8 CNT, 10 AD-, and 10 AD+). Bars represent mean ± SEM. Data were analyzed by Student’s *t*-test in **(A,D,G,H)**, by Pearson’s correlation coefficient in **(B,C,E,F)** and by one-way ANOVA in **(I)** with the Tukey’s test as a *post hoc*. *p = 0.049, **p = 0.0065 when compared with CNT.

As far as we know, the function of the WDFY1 protein in the central nervous system is practically unknown. It has been reported that WDFY1 could be a biomarker for the phenotype severity in the AD11 mouse model of Alzheimer’s disease (Arisi et al., 2011). Additionally, WDFY1 could play a role on TLR3/4 signaling during the differentiation of adult neural stem cells in the dentate gyrus (Yeo et al., 2019). Despite this poor information about the role of WDFY1 in the central nervous system, a previous report where the authors performed a transcriptome outlier analysis placed WDFY1 as a putative upregulated marker in schizophrenia (Duan et al., 2015). Furthermore, in another report the authors showed that WDFY1 could be associated with the DISC1 (from *disrupted in schizophrenia 1*) interactome and regulome (Teng et al., 2018). Thereby we next aimed to verify whether WDFY1 was dysregulated in post-mortem samples from patients with schizophrenia (see Table 1 for sample information). First, we evaluated WDFY1 protein levels in the hippocampal samples and we found that WDFY1 was significantly upregulated (*t*_46_ = 2.845, *p* = 0.0065) in patients with schizophrenia compared to controls (Figure 2A). These changes did not depend on age (Figure 2B) or post-mortem time intervals (Figure 2C). In the case of dorsolateral prefrontal cortex, we also observed a significant upregulation of WDFY1 protein levels (*t*_46_ = 2.018, *p* = 0.049) in schizophrenic patients compared to controls (Figure 2D). Again, these changes did not depend on age (Figure 2E) or post-mortem time intervals (Figure 2F). In contrast, although we observed a trend, WDFY1 protein levels were not significantly upregulated in the putamen of schizophrenic patients compared to controls (Figure 2G). Next, to verify the specificity of these changes, we also evaluated the VSNL1 protein levels in the same hippocampal samples as we did for He^–/–^ mice. Interestingly, VSNL1 protein levels were unchanged in schizophrenic patients compared to controls (Figure 2H). The latter result correlates well with that observed in adult He^–/–^ mice compared with He^+/+^ mice (Figures 1A,B). Finally, since WDFY1 could serve as a biomarker in Alzheimer’s disease (AD), we evaluated WDFY1 protein levels in the hippocampus of AD patients (see Table 2 for sample information) with (AD+) or without (AD-) psychosis compared to controls (Figure 2I). No changes were observed in any subgroup of AD patients compared to controls indicating a possible specificity of this WDFY1 upregulation in patients with schizophrenia but not in patients with AD and psychosis. However, a limitation of the experiment with AD samples was the high variability between samples making its interpretation still a bit inconclusive.

### He^–/–^ Mice Display Altered Impulsive and Dopamine-Associated Behaviors Related to Striatal Dysfunction

**FIGURE 3:**
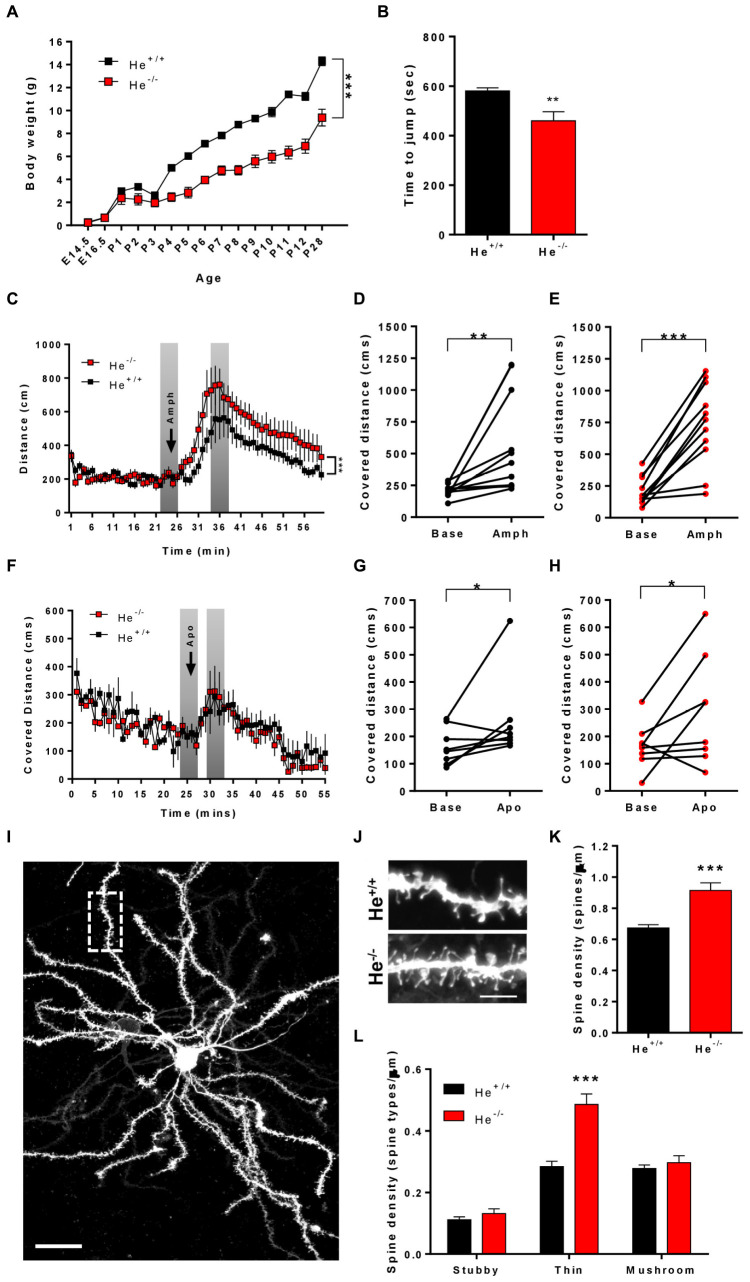
Characterization of schizophrenia-like phenotypes related to striatal function in He^–/–^ mice. **(A)** The curve in graph depicts the body weight gain in both genotypes, He^–/–^ and He^+/+^ mice from embryonic day 14.5 (E14.5) to postnatal day 28 (P28) (*n* = 3 He^+/+^ males and 4 He^+/+^ females, and 4 He^–/–^ males and 4 He^–/–^ females). **(B)** Time to jump out from the glass cylinder in 8-week-old He^–/–^ and He^+/+^ mice (*n* = 4 He^+/+^ males and 4 He^+/+^ females, and 3 He^–/–^ males and 4 He^–/–^ females). **(C)** Locomotor activity in the open field was monitored for 25 min in He^–/–^ and He^+/+^ mice (*n* = 6 He^+/+^ males and 5 He^+/+^ females, and 5 He^–/–^ males and 6 He^–/–^ females). After these 25 min, all mice received an injection of D-amphetamine sulfate (3 mg/kg) as indicated by the top arrow in the graph and the locomotor activity was subsequently monitored for additional 45 min. The induced locomotor activation in He^+/+^ **(D)** and He^–/–^ **(E)** mice was evaluated by comparing representative covered distances from baseline and from treatment as depicted in gray in **(C)**. **(F)** Locomotor activity in the open field was monitored for 25 min in He^–/–^ and He^+/+^ mice (*n* = 8 He^+/+^ and 8 He^–/–^; 4 males an 4 females per genotype). After these 25 min, all mice received an injection of R-(-)-apomorphine (0.5 mg/kg) as indicated by the top arrow in the graph and the locomotor activity was subsequently monitored for additional 45 min. The induced locomotor activation in He^+/+^ **(G)** and He^–/–^ **(H)** mice was evaluated by comparing representative covered distances from baseline and from treatment as depicted in gray in **(F). (I)** Representative images of a DiI-labeled medium spiny neuron (scale bar = 20 microns) and **(J)** representative medium spiny neuron dendrites from 8-weeks-old He*^+/+^* and He^–/–^ mice (scale bar = 3 microns). **(K)** Quantitative analysis showing dendritic spine density per micron of dendritic length from 8-week-old He*^+/+^* and He^–/–^ mice (*n* = 26 dendrites from 5 He^+/+^ mice, 2 males and 3 females, and 20 dendrites from 5 He^–/–^ mice, 3 males and 2 females). **(L)** Density of each type of dendritic spine (stubby, thin, and mushroom) in dendrites of medium spiny neurons from **(K)** in He^–/–^ and He^+/+^ mice. Total evaluated spines: 977 from He^+/+^ mice and 1088 from He^–/–^ mice. Bars represent mean ± SEM. Data were analyzed by unpaired Student’s *t*-test in **(B,K)**, by paired Student’s *t*-test in **(D,E,G,H)** and by two-way ANOVA in **(A,C,F,L)**. **p < 0.01, ***p < 0.001 when compared with He*^+/+^* mice in **(A–C,F,K,L)**. *p < 0.05, **p < 0.01 when compared with baseline data in **(D,E,G,H)**.

Since WDFY1 is up-regulated in the striatum and hippocampus of He^–/–^ mice and in different brain regions of patients with schizophrenia, we then sought to characterize whether He^–/–^ mice would recapitulate some of the schizophrenia-like phenotypes. To deepen on striatal deficiencies related to schizophrenia-like phenotypes, we subjected the 8-week-old He^+/+^ and He^–/–^ mice to the impulsivity test and to treatments with the dopaminergic agonists called amphetamine and apomorphine. First, we noticed that He^–/–^ mice displayed a lower body weight during the entire postnatal development (Figure 3A), which was kept until postnatal day 28 [two-way ANOVA, group effect: *F*_(__1,137__)_ = 477, *p* < 0.0001] as previously described (Cai et al., 2009). Next, we measured impulsivity-like behavior by using the jumping test. In this test, He^–/–^ mice displayed shorter latencies to jump out from the cylinder than He^+/+^ mice (*t*_13_ = 3.297, *p* = 0.0058) suggesting higher levels of impulsivity (Figure 3B). We then intended to observe whether He^–/–^ mice suffer agitation-like behaviors or higher sensitivity to dopaminergic agents. Basal exploratory activity during the first 25 min of free deambulation in the open field was not different between genotypes (Figures 3C,F). As expected, injections with amphetamine- (Figures 3C–E) or with apomorphine-induced (Figures 3F–H) increases in locomotor activity in He^+/+^ (Figure 3D: *t*_10_ = 3.094, *p* = 0.0057, and G: *t*_7_ = 2.045, *p* = 0.0401) and He^–/–^ (Figure 3G: *t*_10_ = 6.522, *p* < 0.001, and H: *t*_7_ = 2.106, *p* = 0.0366) mice. However, the amphetamine- (Figure 3C) but not the apomorphine-induced (Figure 3F) locomotor activity was more pronounced in He^–/–^ mice than in He^+/+^ mice [Two-way ANOVA, group effect after amphetamine injection: *F*_(__59,1175__)_ = 11.67, *p* < 0.001]. We also evaluated microstructural changes in the striatum of 8-week-old He^–/–^ and He^+/+^ mice by analyzing spine density (Figures 3I–K) and morphology (Figure 3L) in medium spiny neurons (MSNs). We found that spine density in MSNs was significantly increased (*t*_43_ = 4.969, *p* < 0.001) in He^–/–^ mice when compared with He^+/+^ mice (Figures 3I–K). Regarding spine morphology (Figure 3L), we detected a clear and specific increase in the density of thin spines in He^–/–^ compared with He^+/+^ mice [Two-way ANOVA, genotype effect: *F*_(__1,129__)_:27.52, *p* < 0.001; Interaction effect: *F*_(__2,129__)_:15.74, *p* < 0.001]. No changes in the density of mushroom or stubby spines were detected between groups (Figure 3L). All results taken together suggest that He^–/–^ mice display a striatal-related psychiatric phenotype.

### He^–/–^ Mice Display Impaired Socio-Cognitive Behaviors Related to Hippocampal Dysfunction

**FIGURE 4:**
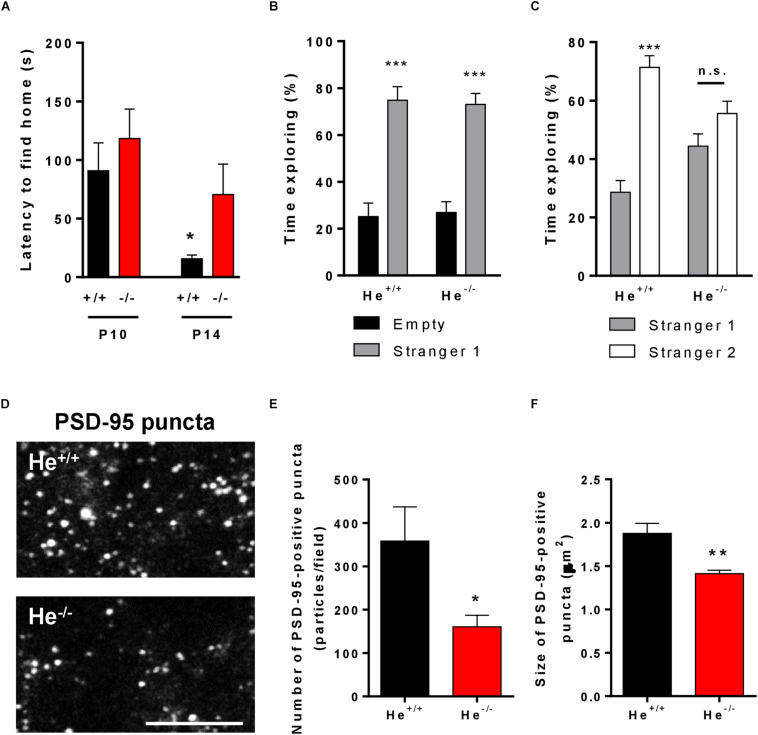
Characterization of schizophrenia-like phenotypes related to hippocampal function in He^–/–^ mice. **(A)** Latency (in seconds) to reach the maternal area in He*^+/+^* and He^–/–^ mice at postnatal day 10 (left, *n* = 3 He^+/+^ males and 6 He^+/+^ females, and 3 He^–/–^ males and 4 He^–/–^ females) and postnatal day 14 (right, *n* = 3 He^+/+^ males and 4 He^+/+^ females, and 3 He^–/–^ males and 3 He^–/–^ females). **(B)** Time exploring the mouse (stranger 1) and empty (empty) cages during socialization in the three-chamber sociability test in 8-week-old He*^+/+^* and He^–/–^ mice (*n* = 6 He^+/+^ and 6 He^–/–^; 3 males and 3 females per genotype). **(C)** Time exploring the known (stranger 1) and the stranger mouse (stranger 2) cages in the social memory and novelty preference evaluation of the three-chamber sociability test in 8-weeks-old He*^+/+^* and He^–/–^ mice. **(D)** Representative inset (17 × 9 microns) of confocal images immunostained for PSD-95 in CA1 *stratum radiatum* (63x objective, digital zoom 5). Original area of analysis per field was 65 × 65 microns. For this experiment, 8-week-old He*^+/+^* and He^–/–^ mice (*n* = 2 He^+/+^ males and 3 He^+/+^ females, and 2 He^–/–^ males and 3 He^–/–^ females) were used. Scale bar = 10 microns. Quantification of the number **(E)** and size **(F)** of PSD-95-positive puncta per field. Bars represent mean ± SEM. Data were analyses by Student’s *t*-test in **(A,E,F)** and by two-way ANOVA with the Bonferroni’s *post hoc* test in **(B,C)**. *p < 0.05, **p < 0.01 when compared with He*^+/+^* mice in **(A,E,F)**. ***p < 0.001 when compared with “empty” in **(B)** or “stranger 1” in **(C)**.

We previously observed that He^–/–^ mice show alterations in spatial learning and memory (Giralt et al., 2019) as occurs in schizophrenic patients (Venkatasubramanian et al., 2014). These deficiencies could be strongly related with a hippocampal dysfunction, a brain region where Helios protein was highly expressed during development (Martín-Ibáñez et al., 2012). We then aimed to evaluate other hippocampal-related phenotypes described to be core symptoms in schizophrenia such as alterations in social behavior (Martinez et al., 2019). We first evaluated maturation of social-cognitive function in pups at early post-natal developmental stages by using the homing test (Alleva and Calamandrei, 1986; Scattoni et al., 2005). The results showed that the homing test latency was significantly lower in He^+/+^ pups at postnatal day 14 (P14) than at P10, indicating a maturation and improvement of the socio-cognitive and neurological functions necessary to solve this task. However, He^–/–^ pups showed no significant improvements in the homing test latencies at P14 compared to P10 and consequently, they showed significantly higher latencies than He^+/+^ pups at P14 (Figure 4A, *t*_11_ = 2.267, *p* = 0.0445). To further confirm these social deficiencies, we performed the three-chamber social interaction test in 8-week-old He^–/–^ and He^+/+^ mice. In a first phase, He^–/–^ mice showed normal sociability (Figure 4B). However, in a second phase He^–/–^ mice showed decreased social memory and social novelty preference [Two-way ANOVA, group effect: *F*_(__1,20__)_ = 43.75, *p* < 0.0001] when compared with He^+/+^ mice (Figure 4C).

We next aimed to explore putative changes in excitatory synapses associated with spine alterations previously reported in the pyramidal neurons of the He^–/–^ mice (Giralt et al., 2019). Such changes could have a potential correlation with their deficits in social skills. We evaluated the postsynaptic marker called PSD-95 previously reported to be decreased in the hippocampal CA1 of schizophrenic patients (Matosin et al., 2016). Thereby, we analyzed this marker in the CA1 *stratum radiatum* of 8-week-old He^+/+^ and He^–/–^ mice. We observed that the number of PSD-95-positive puncta was significantly decreased (*t*_8_ = 2.37, *p* = 0.0453) in He^–/–^ compared with He^+/+^ mice (Figures 4D,E). Furthermore, the size of the PSD-95-positive puncta was also significantly reduced (*t*_8_ = 3.768, *p* = 0.005) in He^–/–^ mice with respect to He^+/+^ mice (Figure 4F). In summary, these results suggest that the social deficiencies observed in He^–/–^ mice compared with He^+/+^ mice were accompanied by changes in the number and size of PSD-95-positive clusters in the apical dendrites of the CA1.

### NF-κB Levels Are Reduced in the Neuronal Soma of CA1 Pyramidal Neurons in He^–/–^ Mice

**FIGURE 5:**
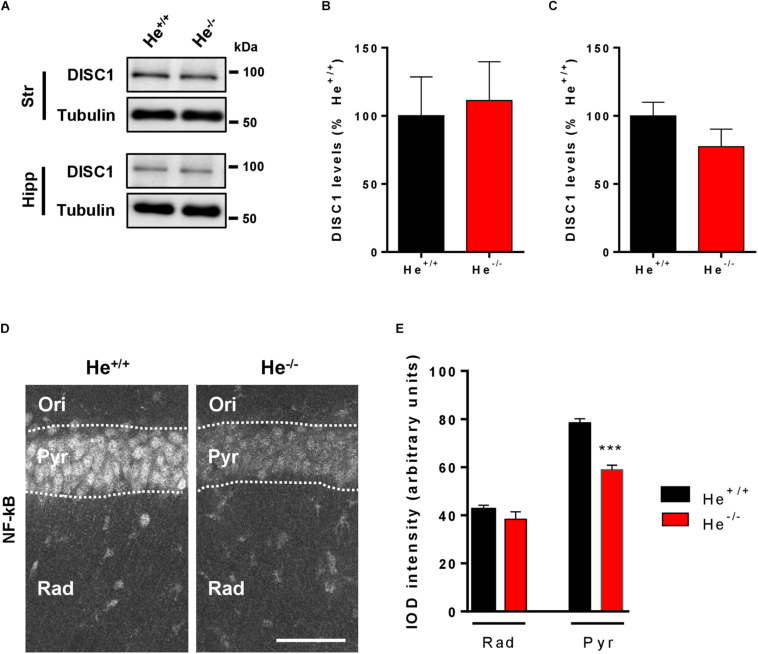
Levels and distribution of DISC1 and NF-κB in the hippocampus of He^–/–^ mice. **(A)** Immunoblotting for DISC1 and Tubulin as a loading control in the striatum (Str, *n* = 9/genotype) or hippocampus (Hipp, *n* = 10–11/genotype) of 8-week-old He^+/+^ and He^–/–^ mice. Densitometry quantification in the striatal **(B)** and hippocampal **(C)** samples of results as in **(A)**. Data were normalized to tubulin for each sample and expressed as percentage of wild type. **(D)** Representative confocal images of NF-κB immunofluorescence in CA1 (40x objective) of 8-week-old He*^+/+^* and He^–/–^ mice (*n* = 10 He^+/+^ and 8 He^–/–^). Scale bar = 70 microns. **(E)** Quantification of NF-κB the IOD per layer (total IOD mean per layer). Bars represent mean ± SEM. Data were analyzed by Student’s *t*-test. ***p < 0.001 when compared with He*^+/+^* mice in **(E)**.

Since WDFY1 increased levels were observed in the hippocampus of both schizophrenic patients and 8-week-old He^–/–^ mice, we then evaluated the levels of DISC1 and the distribution of NF-κB in this brain region. We selected these molecular targets because DISC1 is a well-known genetic risk factor for schizophrenia (Porteous et al., 2014) and it has been shown to be a target of TLR3/4 downstream signaling (Chen et al., 2017). NF-κB was evaluated because the TLR3/4-dependent regulation of NF-κB is potentiated when WDFY1 is over-expressed (Hu et al., 2015) and NF-κB is a core neuroinflammatory molecule involved in the pathogenesis of schizophrenia (Dwir et al., 2019). First, we observed that DISC1 protein levels were unchanged in the striatum and in the hippocampus of 8-week-old He^–/–^ mice compared to He^+/+^ litters (Figures 5A–C). Then, we evaluated by immunofluorescence the levels and distribution of NF-κB (p65) in the CA1 of He^–/–^ and He^+/+^ mice (Figure 5D). Unexpectedly, we found a significantly reduced NF-κB (p65) expression (*t*_16_ = 7.772, *p* = 0.001), specifically in the pyramidal cell layer of the dorsal hippocampus in He^–/–^ mice compared with age-matted He^+/+^ litters (Figures 5D,E). These results suggest a connection between the increase on WDFY1 levels and the reduction of NF-κB in the hippocampus of He^–/–^ mice.

### Pharmacological Models of Schizophrenia Do Not Induce Changes in WDFY1 Protein Levels

**FIGURE 6:**
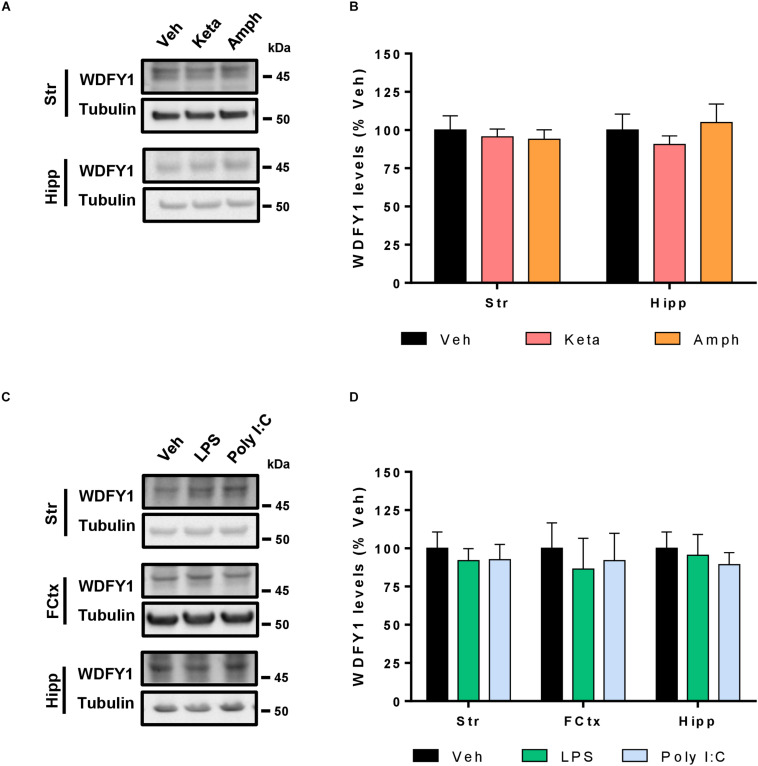
WDFY1 protein levels in pharmacological models of schizophrenia. **(A)** Immunoblotting for WDFY1 and Tubulin as a loading control in the striatum (Str) and hippocampus (Hipp) of 10-week-old C57BL/6 mice treated with vehicle or amphetamine (3 mg/kg) or ketamine (30 mg/kg) for 8 days (*n* = 8 vehicle- (Veh), 10 ketamine- (Keta) and 11 amphetamine-treated (Amph) mice). **(B)** Densitometry quantification of results as in **(A)**. Data were normalized to tubulin for each sample and expressed as percentage of wild type. **(C)** Immunoblotting for WDFY1 and Tubulin as a loading control in the striatum (Str), frontal cortex (FCtx) and hippocampus (Hipp) of C57BL/6 mice treated at postnatal day 5 with vehicle or LPS (6 mg/kg) or Poly I:C (6 mg/kg) and samples collected 24 h later (*n* = 9 vehicle- (Veh), 6 LPS- and 7 Poly I:C-treated mice). **(D)** Densitometry quantification of results as in **(C)**. Data were normalized to tubulin for each sample and expressed as percentage of wild type. Bars represent mean ± SEM. Data were analyzed by one-way ANOVA and the Tukey’s test as a *post hoc* test in all panels.

As we have seen increased WDFY1 protein levels in the hippocampus and striatum of He^–/–^ mice and in the hippocampus and dorsolateral prefrontal cortex in schizophrenic post-mortem samples, we then hypothesized that pharmacological models of schizophrenia could mimic such molecular changes. First, we treated 10-week-old male wild-type mice with vehicle or amphetamine (3 mg/kg) or ketamine (30 mg/kg) for 8 days. All mice were sacrificed 15 min after last injection on day 8. Striatal and hippocampal samples from these mice were evaluated by western blot to check for the WDFY1 protein levels. Our results indicate that WDFY1 protein levels are not influenced by any of both treatments in any brain region (Figures 6A,B). We then hypothesized that pharmacological treatments aimed to induce a strong and persistent neuroinflammatory response during neurodevelopmental stages could mimic the changes on WDFY1 protein levels as we observed in He^–/–^ mice and human post-mortem samples from schizophrenic patients. Therefore, we treated wild-type mouse pups with vehicle or lipopolysaccharides (LPS, 6 mg/kg) or Poly I:C (6 mg/kg) at postnatal day 5 and collected striatal, hippocampal, and frontal cortex samples 24 h later. Again, western blot experiments demonstrated that WDFY1 protein levels are not influenced by any of both treatments in any brain region analyzed (Figures 6C,D). Taken together, these results indicate that pharmacological models of schizophrenia that model dopaminergic- (amphetamine) or glutamatergic-related (ketamine) pathophysiological events or neurodevelopmental immunological challenges (LPS or Poly I:C respectively) do not recapitulate the changes on WDFY1 protein levels observed in He^–/–^ mice and schizophrenic patients.

## Discussion

In the present work we show that mice devoid of Helios (He^–/–^ mice) recapitulate negative schizophrenic-like symptoms classically modeled in rodents, namely increased impulsivity, amphetamine-induced locomotor activity, and decreased social skills. Here we also report an increased spine density in MSNs and decreased excitatory PSD-95 positive clusters in pyramidal neurons of the CA1 in He^–/–^ mice. All these deficiencies correlate with a sustained and specific increase on WDFY1 mRNA levels in the striatum and hippocampus. We also showed increased WDFY1 protein levels in the CA1 but not in the striatum. Further future studies will be necessary to confirm the WDFY1 protein levels in the striatum with a potential predicted increase as we observed when mRNA levels were analyzed. Accordingly, postmortem samples from schizophrenic patients but not from Alzheimer’s disease patients also display an increase of WDFY1 protein levels in the hippocampus and in the dorso-lateral prefrontal cortex, whereas in He^–/–^ mice we found an increase of WDFY1 in the hippocampus and striatum. Therefore, the hippocampus is the only brain region that commonly displayed increased WDFY1 levels in both, schizophrenic patients and He^–/–^ mice.

First, we found that He^–/–^ mice displayed a remarkable lower body weight detected almost since they were born, a variable that was never recovered in adult mice. Low birth weight has been strongly associated with schizophrenia and with worse social and cognitive abilities in these patients (Rifkin et al., 1994). Lower body weight has also been described even in adult patients until they suffer the first episode (Wetterling et al., 2004). In contrast, after this first episode, schizophrenic patients suffer a remarkable increase of body weight (Holt et al., 2018) due, at least in part, to chronic antipsychotic treatments (Baptista, 1999). Regarding to the striatal-dependent alterations, we previously observed that He^–/–^ mice show subtle alterations in motor skills (Martín-Ibáñez et al., 2017) as occurs in schizophrenic patients (Wolff and O’Driscoll, 1999), which was related to an altered striatal function, although a role of the cerebellum should not be ruled out. Here we have increased such previous data by describing an aberrant impulsive behavior associated with an increase of the spine density in MSNs in the same line as occurs with schizophrenic patients (Glausier and Lewis, 2013; Hoptman, 2015). Surprisingly, He^–/–^ mice did show increased agitation (locomotor activity) upon amphetamine injection but not upon apomorphine injection, suggesting that Helios deficiency could be preferentially involved with negative symptomatology and just moderately with positive symptomatology (Powell and Miyakawa, 2006) or alternatively, that Helios deficiency partially impacts the dopaminergic system. Finally, it is noteworthy that the observed increase of spine density in MSNs in He^–/–^ mice also occurs in schizophrenic patients and correlate with negative and cognitive symptoms (Roberts et al., 2005, 2008).

Regarding the hippocampal-related phenotype, we recently described that He^–/–^ mice display hippocampal-dependent spatial learning and memory impairments associated with alterations in synaptic plasticity (Giralt et al., 2019). The findings described here include decreased density and size of PSD-95 clusters in the CA1, altered maternal-filial social behavior, and severe social novelty/memory impairments in He^–/–^ mice. Decreased PSD-95 levels have been described in the CA1 of schizophrenic patients (Matosin et al., 2016). Indeed, decreased PSD-95 levels is one of the most consistent synaptic alterations in the hippocampus from patients as recently summarized in a broad metanalysis (Osimo et al., 2019). Patients with schizophrenia also show consistent deficits in spatial working memory and social skills (Brüne et al., 2011). Such alterations could be perfectly associated to an hippocampal dysfunction since this brain region modulates socio-cognitive skills and spatial memory (Shrager et al., 2007; Rubin et al., 2014). In line with this, it is noteworthy that the hippocampus is morphologically the most affected brain region in schizophrenia (Mancini et al., 2019).

A potential mechanism of the schizophrenia-like phenotype observed in He^–/–^ mice could be the sustained increase on WDFY1 levels in different core brain regions implicated in schizophrenia. First, because it is the only gene that remains altered in different brain regions from the RNAseq experiment performed in our previous study at embryonic day 18 (Giralt et al., 2019). Second, because WDFY1 is upregulated in the hippocampus and dorso-lateral prefrontal cortex of patients with schizophrenia and in the hippocampus and striatum of He^–/–^ mice. Although our pharmacological treatments with LPS and Poly I:C do not induce changes on WDFY1 levels, we hypothesize that the sustained increase of WDFY1 levels in He^–/–^ mice and schizophrenia patients could be due to an impairment of a proper neuroimmunomodulation that may have its origin in the early phases of the neurodevelopment, when Helios is specifically expressed (Martín-Ibáñez et al., 2012, 2017). The hypothesized impairment in the neuroimmunomodulation could be provoked by molecular changes involving the Helios-dependent pathway. The fact that Helios is a potent regulator of molecular pathways related to immunity (Heizmann et al., 2018) strengthens our hypothesis.

In this line, since we detected a persistent upregulation of WDFY1 in the CA1 pyramidal cells of He^–/–^ mice, we expected a resulting increase on neuronal NF-κB expression (Hanke and Kielian, 2011; Okun et al., 2011) as previously described in other cell types (Hu et al., 2015). Conversely, we observed a reduced NF-κB (p65) expression in the cell bodies/nuclei of these CA1 pyramidal cells, which could suggest a decrease in the ability of NF-κB to activate transcription. There could be some potential explanations for these apparently contradictory results. First, in the previous work by Hu et al. (2015) the authors observed in 293-TLR3 cells a potentiated NF-κB activation by WDFY1 only in stimulation conditions of the TLR3/4 receptors but not in basal conditions. The proposed mechanism was the recruitment of TRIF by WDFY1. Thereby, we hypothesize that increased WDFY1 levels without immunologic stimulation (present results) does not necessarily potentiate the NF-κB signaling/levels. Second, the dynamics and functions of the WDFY1:NF-κB pathway is completely unknown in neuronal cells and the potential interactors with WDFY1 in neurons is an open field to deepen on in future studies. Thus, it is widely accepted that in peripheral or in glial cells, NF-κB activation induces inflammatory or pro-apoptotic pathways and responses via nuclear translocation as a main but not the only mechanism (Liu et al., 2017). However, the role of NF-κB in neurons seems to be more oriented to the modulation of synaptic plasticity and cognitive processes such as learning and memory (Meffert et al., 2003; Dresselhaus and Meffert, 2019). Furthermore, NF-κB is a main transcriptional inductor of PSD-95 expression (Boersma et al., 2011) which is consistent with our findings of the localized reduction of the scaffold protein in the CA1 of mice devoid of Helios. Finally, although some previous studies have shown that NF-κB is increased in peripheral cells of schizophrenic patients (Song et al., 2009), studies evaluating brain post-mortem samples showed a significant decrease of NF-κB expression in cerebellum (MacDowell et al., 2017), dorsolateral prefrontal cortex, and temporal area gyrus (Roussos et al., 2013). Altogether, these results indicate a decrease in the ability of NF-κB to activate transcription and are in accordance with different potential roles and distinct mechanisms of regulation of NF-κB depending on the tissue to be analyzed and, in turn, strengthen our hypothesis that reduced NF-κB in CA1 pyramidal neurons could play a role in the schizophrenic-like phenotype observed in He^–/–^ mice.

Yet our study has some limitations. First, the finding that the *Wdfy1* gene is upregulated in the hippocampus of He^–/–^ mice could be indirect since no direct evidence of transcription control of *Wdfy1* by Helios has been previously observed in literature using microarrays or Chip-seq approaches (Kim et al., 2015; Zhao et al., 2016) in tissues other than nervous system. Similar lack of direct evidence was obtained when searching in public databases such as GEO^1^ or Harmonizome^2^. In line with this, although in the present manuscript we have analyzed all the genes differentially expressed in the RNAseq experiment from our previous study (Giralt et al., 2019) and we found that only *Wdfy1* was still upregulated, our previous RNAseq was performed in embryonic hippocampal tissue at E18 (Giralt et al., 2019). As a consequence, we cannot rule out that other unexplored genes are differentially expressed in the adult neural tissue of He^–/–^ mice that could also be involved with the psychiatric disturbances described here. Finally, although Helios (Martín-Ibáñez et al., 2012, 2017; Giralt et al., 2019) and WDFY1 (present results^3^) are enriched in neurons, increased WDFY1 levels could exert an alternative cell-non-autonomous effect since TLR receptors are mostly expressed in glia (Kielian, 2006) whereas they are expressed in a lesser extent in neurons (Hanke and Kielian, 2011; Okun et al., 2011).

Taken altogether, we propose the He^–/–^ mice as a model to study how alterations during the development of the central nervous system could account for molecular pathways typically involved with immunomodulatory processes that would later precipitate the appearance of neuropsychiatric disorders such as schizophrenic pathology.

## Data Availability Statement

All datasets generated for this study are included in the article/supplementary material.

## Ethics Statement

The animal study was reviewed and approved by CEEA-UB (Comitè Ètic d’Experimentació Animal de la Universitat de Barcelona).

## Author Contributions

AS-B performed western blot, samples organization and management, immunofluorescence, behavioral experiments, and helped to write the manuscript. BF performed western blot and immunofluorescence. VB performed the DioListic experiments and spine counting. MP performed behavioral experiments. MS performed the mRNA extraction and Q-PCR experiments and subsequent analysis. JA helped to write the manuscript. IH diagnosed the Alzheimer’s disease patients (with or without psychosis) and helped in the organization and the collection and processing of the AD post-mortem samples. BA performed the organization of the schizophrenic post-mortem samples, its collecting and processing procedures, and helped write the manuscript. JC helped write the manuscript. AG wrote the manuscript, thought all the experimental design, and performed some behavioral experiments. SG helped to write the manuscript and with the design of the behavioral experiments.

## Conflict of Interest

The authors declare that the research was conducted in the absence of any commercial or financial relationships that could be construed as a potential conflict of interest.

## Footnotes

1
https://omictools.com/a1ae9227ffba28725cba03e44b02aee1-dataset

2
http://amp.pharm.mssm.edu/Harmonizome/gene/WDFY1

3
https://mouse.brain-map.org/gene/show/45210
