# Corrigendum: Lack of Helios during neural development induces adult schizophrenia-like behaviors associated with aberrant levels of the TRIF-recruiter protein WDFY1

**DOI:** 10.3389/fncel.2024.1542681

**Published:** 2025-01-07

**Authors:** Anna Sancho-Balsells, Veronica Brito, Belissa Fernández, Mónica Pardo, Marco Straccia, Silvia Ginés, Jordi Alberch, Isabel Hernández, Belén Arranz, Josep M. Canals, Albert Giralt

**Affiliations:** ^1^Departament de Biomedicina, Facultat de Medicina i Ciències de la Salut, Universitat de Barcelona, Barcelona, Spain; ^2^Institut de Neurociències, Universitat de Barcelona, Barcelona, Spain; ^3^Institut d'Investigacions Biomèdiques August Pi i Sunyer (IDIBAPS), Barcelona, Spain; ^4^Centro de Investigación Biomédica en Red sobre Enfermedades Neurodegenerativas (CIBERNED), Madrid, Spain; ^5^Laboratory of Stem Cells and Regenerative Medicine, Department of Biomedical Sciences, Faculty of Medicine and Health Science, University of Barcelona, Barcelona, Spain; ^6^Faculty of Medicine and Health Science, Production and Validation Center of Advanced Therapies (Creatio), University of Barcelona, Barcelona, Spain; ^7^Alzheimer Research Center and Memory Clinic, Fundació ACE, Institut Català de Neurociències Aplicades. Barcelona, Spain; ^8^Parc Sanitari Sant Joan de Déu, CIBERSAM, Barcelona, Spain

**Keywords:** hippocampus, putamen, cortex, FENS1, psychosis, negative symptoms, DISC1

In the published article, there was an error in the image used for Figure 3J as published. The corrected Figure 3J and its caption appear below.

**Figure 3:**
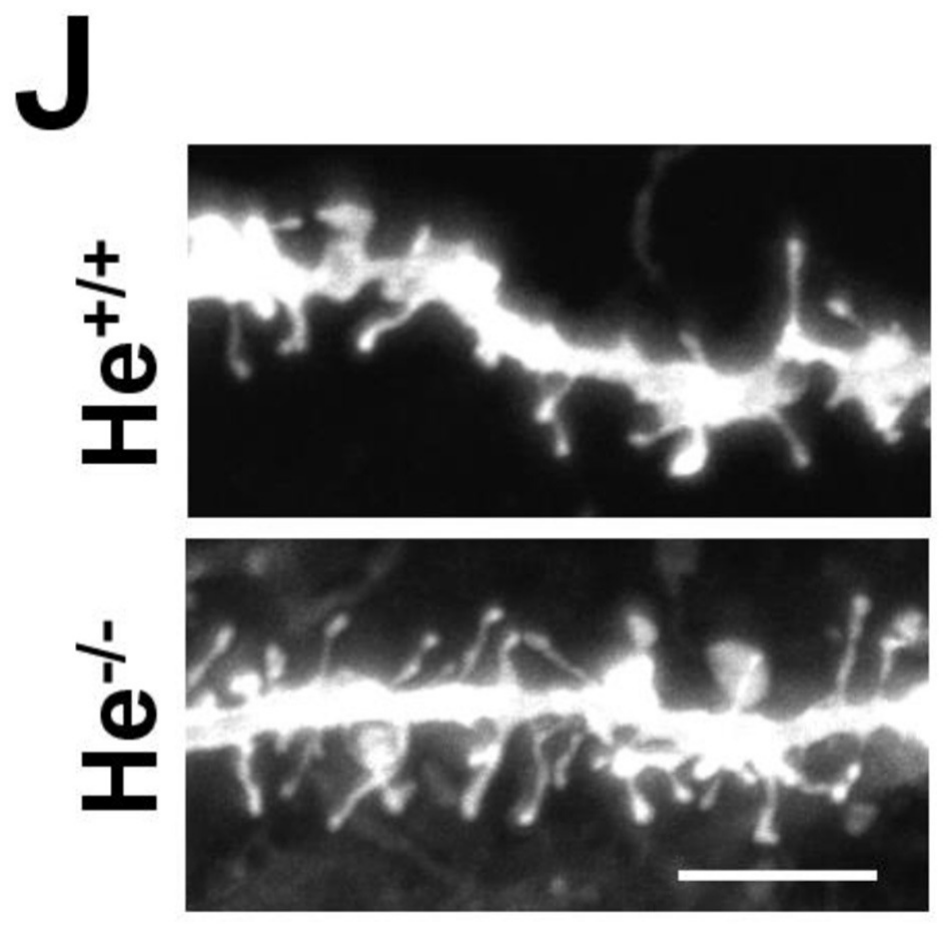
Characterization of schizophrenia-like phenotypes related to striatal function in He–/– mice. **(A)** The curve in graph depicts the body weight gain in both genotypes, He–/– and He+/+ mice from embryonic day 14.5 (E14.5) to postnatal day 28 (P28) (*n* = 3 He+/+ males and 4 He+/+ females, and 4 He–/– males and 4 He–/– females). **(B)** Time to jump out from the glass cylinder in 8-week-old He–/– and He+/+ mice (*n* = 4 He+/+ males and 4 He+/+ females, and 3 He–/– males and 4 He–/– females). **(C)** Locomotor activity in the open field was monitored for 25 min in He–/– and He+/+ mice (*n* = 6 He+/+ males and 5 He+/+ females, and 5 He–/– males and 6 He–/– females). After these 25 min, all mice received an injection of D-amphetamine sulfate (3 mg/kg) as indicated by the top arrow in the graph and the locomotor activity was subsequently monitored for additional 45 min. The induced locomotor activation in He+/+ **(D)** and He–/– **(E)** mice was evaluated by comparing representative covered distances from baseline and from treatment as depicted in gray in **(C)**. **(F)** Locomotor activity in the open field was monitored for 25 min in He–/– and He+/+ mice (*n* = 8 He+/+ and 8 He–/–; 4 males an 4 females per genotype). After these 25 min, all mice received an injection of R-(-)-apomorphine (0.5 mg/kg) as indicated by the top arrow in the graph and the locomotor activity was subsequently monitored for additional 45 min. The induced locomotor activation in He+/+ **(G)** and He–/– **(H)** mice was evaluated by comparing representative covered distances from baseline and from treatment as depicted in gray in **(F)**. **(I)** Representative images of a DiI-labeled medium spiny neuron (scale bar = 20 microns) and **(J)** representative medium spiny neuron dendrites from 8-weeks-old He+/+ and He–/– mice (scale bar = 3 microns). **(K)** Quantitative analysis showing dendritic spine density per micron of dendritic length from 8-week-old He+/+ and He–/– mice (*n* = 26 dendrites from 5 He+/+ mice, 2 males and 3 females, and 20 dendrites from 5 He–/– mice, 3 males and 2 females). **(L)** Density of each type of dendritic spine (stubby, thin, and mushroom) in dendrites of medium spiny neurons from **(K)** in He–/– and He+/+ mice. Total evaluated spines: 977 from He+/+ mice and 1088 from He–/– mice. Bars represent mean ± SEM. Data were analyzed by unpaired Student's *t-*test in **(B, K)**, by paired Student's t-test in **(D, E, G, H)** and by two-way ANOVA in **(A, C, F, L)**. ^**^p < 0.01, ^***^p < 0.001 when compared with He+/+ mice in **(A–C, F, K, L)**. ^*^p < 0.05, ^**^p < 0.01 when compared with baseline data in **(D, E, G, H)**.

The authors apologize for this error and state that this does not change the scientific conclusions of the article in any way. The original article has been updated.

